# Novel Mutation in *CYP2R1* Causing Vitamin D-Dependent Rickets Type 1b

**DOI:** 10.1210/jcemcr/luae024

**Published:** 2024-03-04

**Authors:** Jayakrishnan C Menon, Archana Kumari, Shruti M Sajjan, Preeti Dabadghao

**Affiliations:** Department of Endocrinology, Sanjay Gandhi Postgraduate Institute of Medical Sciences, Lucknow, Uttar Pradesh 226014, India; Department of Endocrinology, Sanjay Gandhi Postgraduate Institute of Medical Sciences, Lucknow, Uttar Pradesh 226014, India; Department of Endocrinology, Sanjay Gandhi Postgraduate Institute of Medical Sciences, Lucknow, Uttar Pradesh 226014, India; Department of Endocrinology, Sanjay Gandhi Postgraduate Institute of Medical Sciences, Lucknow, Uttar Pradesh 226014, India

**Keywords:** VDDR1b, *CYP2R1*, vitamin D dependent rickets, resistant rickets, monogenic rickets

## Abstract

Monogenic forms of rickets are being increasingly recognized. However, vitamin D-dependent rickets 1b (VDDR1b) due to *CYP2R1* gene mutation is exceedingly rare. We report a 4.5-year-old girl and her younger sibling who presented with clinical, radiological, and biochemical features suggestive of nutritional rickets that did not resolve despite repeated therapeutic doses of vitamin D3. This led to evaluation for resistant rickets, which revealed a novel homozygous *CYP2R1* c.50_51insTCGGCGGCGC; p.Leu18ArgfsTer79 variant in the affected siblings. The children were treated with oral calcium and cholecalciferol, dose titrated to maintain serum alkaline phosphatase, 25 hydroxy vitamin D, and parathyroid hormone levels in the normal range, with good clinical and radiological response. This case highlights the importance of genetic evaluation in patients with suspected nutritional rickets who have a family history of similar illness and require higher than usual doses of vitamin D for healing or relapse on stopping treatment. To the best of our knowledge this is the first case of VDDR1b reported from Asia.

## Introduction

Rickets has traditionally been considered a manifestation of poor socioeconomic status and the presence of dietary and social factors that fail to provide adequate amounts of calcium and/or vitamin D. However, as society has progressed and awareness about the condition has increased, rare genetic defects in vitamin D metabolism are increasing in prominence.

Vitamin D-dependent rickets (VDDR) refers to a group of genetic disorders characterized by the inability to maintain adequate concentrations of active forms of vitamin D or a failure to respond fully to active vitamin D (1). VDDR type 1 represents a failure to fully activate calciferols due to the inability to generate either 25 hydroxy vitamin D (25OHD) (VDDR1b) or 1,25 dihydroxy vitamin D [1,25(OH)_2_D] (VDDR1a).

VDDR1b has been established to be due to mutations in the *CYP2R1* gene. The condition is rare with only 7 known mutations reported in literature to date (2‐7). Here we report the clinical and biochemical features of a family with a novel mutation in *CYP2R1,* which represents the first report of the condition from our geographical area.

## Case Presentation

**Figure 1.**
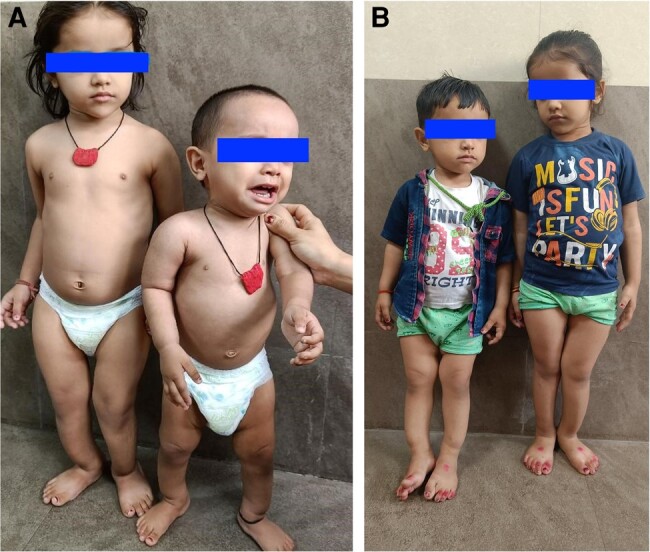
Clinical picture of the siblings (A) at first visit and (B) after 6 months of therapy. Note the slight improvement in the right lower limb for the girl child (Proband).

A 4-year 10-month-old girl child, born of a nonconsanguineous marriage, presented to our institution with complaints of waddling gait and bowing of legs noticed since 16 months of age. She had no history of convulsions, tetany, alopecia, dental abnormalities, or hearing defects. There was no history suggestive of renal tubular defects, malabsorption, or other systemic causes of resistant rickets. She had received therapeutic doses of vitamin D multiple times at the age of 16 months, 3 years, and 4.5 years with incomplete response. Her younger brother (age 2 years) also had similar complaints since the age of 12 months. There was no history of similar illness in other members of her extended family. Birth and developmental history were normal. Her daily calcium intake was 400 mg, mainly from dairy products. Sun exposure was inadequate. On examination, her weight was 11 kg (−2.87 SD), height was 88 cm (−3.37 SD), and upper segment: lower segment ratio was 1.4. She had stigmata of rickets in the form of frontal bossing, widening of wrists, Harrison's sulcus, anteriorly bent legs, and double malleoli. Systemic examination was within normal limits (Table 1 and Fig. 1).

**Table 1.** Biochemical parameters and follow-up of the proband and her family

Parameters	Proband (outside hospital)	Younger sibling (outside hospital)	Proband (1st visit)	Younger sibling (1st visit)	Proband (after 3 months*^a^*)	Younger sibling (after 3 months*^a^*)	Proband (after 9 months*^a^*)	Younger sibling (after 9 months*^a^*)	Mother	Father	Elder sibling
Age/sex	4y 10 m/F	3 y/M	4 y 10 m/M	3 y/M	5 y 1 m/F	3 y 3 m/M	5 y 7 m/F	3 y 9 m/M	31 y	36 y	8 y
Mutation	HM	HM	HM	HM	HM	HM	HM	HM	HT	HT	WT
Clinical presentation	Florid rickets with Genu valgum deformity Waddling gait	Florid rickets with Genu valgum deformity with multiple fracture Waddling gait	Gait improved Deformities persisted	Gait improved Deformities persisted	Further improvement in gait and deformities	Further improvement in gait and deformities	Further improvement in gait and deformities	Further improvement in gait and deformities	No complaints	No complaints	No complaints
S calcium	8.4 mg/dL (2.1 mmol/L)	9.3 mg/dL (2.3 mmol/L)	9.4 mg/dL (2.4 mmol/L)	9 mg/dL (2.3 mmol/L)	9.3 mg/dL (2.3 mmol/L)	9.7 mg/dL (2.4 mmol/L)	9.6 mg/dL (2.4 mmol/L)	10.2 mg/dL (2.6 mmol/L)	9.1 mg/dL (2.3 mmol/L)	8.6 mg/dL (2.2 mmol/L)	10.1 mg/dL (2.5 mmol/L)
S phosphorus^*b*^	3.5 mg/dL (1.13 mmol/L)	4.9 mg/dL (1.58 mmol/L)	5.1 mg/dL (1.65 mmol/L)	5 mg/dL (1.61 mmol/L)	3.8 mg/dL (1.23 mmol/L)	5.2 mg/dL (1.68 mmol/L)	NA	NA	2.5 mg/dL (.81 mmol/L)	2.7 mg/dL (.87 mmol/L)	4.5 mg/dL (1.45 mmol/L)
ALP^*c*^ (IU/L)	1393	2178	297	565	278	335	288	216	41	57	287
25OHD	3.3 ng/mL (8.3 nmol/L)	80 ng/mL (200 nmol/L)	107.2 ng/mL (268 nmol/L)	120 ng/mL (300 nmol/L)	17.2 ng/mL (43 nmol/L)	20.5 ng/mL (51.3 nmol/L)	20.1 ng/mL (50.3 nmol/L)	23.6 ng/mL (59 nmol/L)	10.9 ng/mL (27.3 nmol/L)	17.3 ng/mL (43.3 nmol/L)	13.5 ng/mL (33.8 nmol/L)
PTH	268 pg/mL (28.4 pmol/L)	NA	36 pg/mL (3.8 pmol/L)	190 pg/mL (20.2 pmol/L)	NA	NA	36 pg/mL (3.8 pmol/L)	79 pg/mL (8.4 pmol/L)	96 pg/mL (10.2 pmol/L)	66 pg/mL (7.0 pmol/L)	44.7 pg/mL (4.7 pmol/L)
Radiology	Metaphysealcupping, fraying, and splaying	Metaphysealcupping, fraying, and splaying	Minimum line of healing noted	Minimum line of healing noted	Line of healing noted	Line of healing noted	Healed X-ray	Healed X-ray	NA	NA	NA
Treatment	Approximately 6 lac IU 25OHD oral + calcium 500 mg	Approximately 6 lac IU 25OHD oral + calcium 500 mg	All treatment stopped since toxic levels achieved	All treatment stopped since toxic levels achieved	Cholecalciferol @ 2000 IU/day + calcium 500 mg	Cholecalciferol @ 2000 IU/day + calcium 500 mg	Cholecalciferol @ 4000 IU/day + calcium 500 mg	Cholecalciferol @ 4000 IU/day + calcium 500 mg	Cholecalciferol @ 300 000 units bolus	Cholecalciferol @ 300 000 units bolus	Cholecalciferol @ 300 000 units bolus

^*a*^After first visit to our hospital.

^*b*^Reference range for serum phosphorus varies with age, as follows:

• 0-5 days—4.8-8.2 mg/dL (1.55-2.65 mmol/L)

• 1-3 years—3.8-6.5 mg/dL (1.23-2.10 mmol/L)

• 4-11 years—3.7-5.6 mg/dL (1.19-1.81 mmol/L)

• 12-15 years—2.9-5.4 mg/dL (.94-1.74 mmol/L)

• >15 years—2.7-4.7 mg/dL (.87-1.52 mmol/L)

^*c*^The reference range for serum ALP is <150 IU/L for adults, in our assay. The upper limit of normal in children < 12 years of age is approximately 3 times the upper limit in adults.

Normal reference ranges: calcium—8.5-10.8 mg/dL (2.1-2.7 mmol/L); 25OHD—20-100 ng/mL (50-250 nmol/L); iPTH—15-65 pg/mL (1.6-6.9 pmol/L).

Abbreviations: 25OHD, 25 hydroxy vitamin D; ALP, alkaline phosphatase; HM, homozygous; HT, heterozygous; iPTH, intact parathyroid hormone; NA, not available; PTH, parathyroid hormone; WT, wild type.

*^a^* After first visit to our hospital.

## Diagnostic Assessment

**Figure 2.**
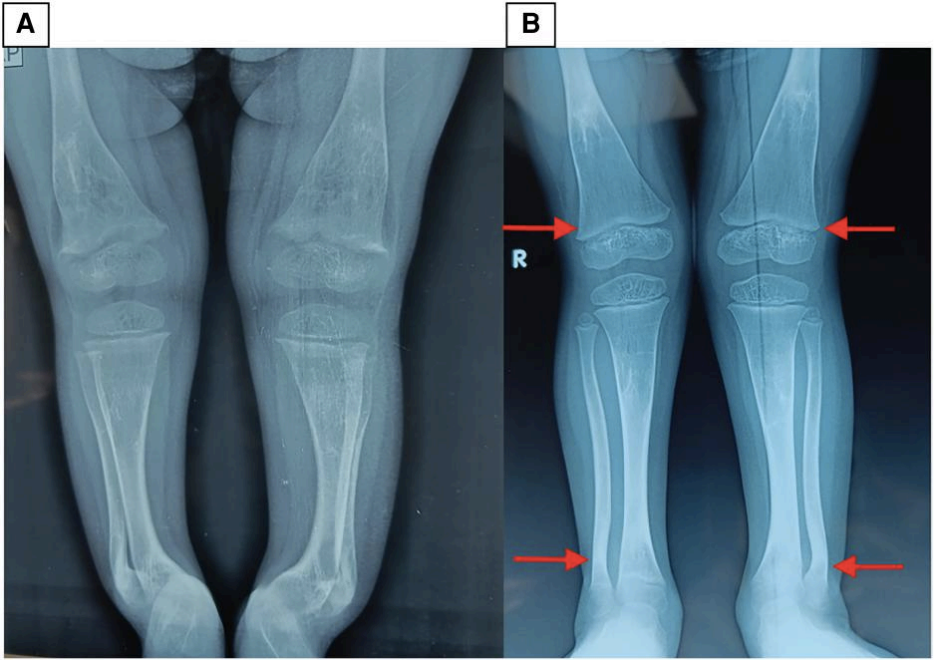
X-rays of the proband before (A) and after (B) treatment. X-ray of bilateral lower limbs of the proband before (A) and after (B) treatment with cholecalciferol. Panel A shows metaphyseal fraying, splaying, and cupping of the lower femoral metaphysis and anterolateral bowing in bilateral lower tibia and fibula. Panel B shows resolution of the metaphyseal changes and improvement in deformity in the tibia and fibula (arrows).

Investigations done in October 2021 prior to presentation showed serum calcium 8.4 mg/dL (2.1 mmol/L) (normal reference range: 8.5-10.8 mg/dL; 2.1-2.7 mmol/L), serum phosphate 3.5 mg/dL (1.13 mmol/L) (normal reference range for age: 3.7-5.6 mg/dL; 1.19-1.81 mmol/L), serum alkaline phosphatase (ALP) 1393 U/L (normal reference range in adults: < 150), serum 25OHD 3.3 ng/mL (8.25 nmol/L) (normal reference range: 20-100 ng/mL; 50-250 nmol/L), and serum intact parathyroid hormone (iPTH) 268 pg/mL (28.8 pmol/L) (normal reference range: 15-65 pg/mL; 1.6-6.9 pmol/L). Plain radiographs of the lower limbs showed changes of florid rickets (Fig. 2). She was subsequently given 60 000 IU cholecalciferol weekly for 10 doses and was referred to our center. Initial evaluation in our center in May 2022 showed serum calcium 9.4 mg/dL (2.35 mmol/L), serum phosphorus 5.1 mg/dL (1.65 mmol/L), serum ALP 297 IU/L, serum 25OHD 107.2 ng/mL (268 nmol/L), and serum iPTH 34 pg/mL (3.6 pmol/L). Since 25OHD levels were elevated, we stopped vitamin D supplementation and monitored the patient. Reevaluation in August 2022 after 2 months showed that serum 25OHD levels had rapidly fallen to 17 ng/mL (43 nmol/L). Serum calcium, phosphorus, and ALP were within the normal range for age. X-rays of the wrist and lower limbs showed evidence of healing (Fig. 2).

In view of the early age of onset, inadequate response to treatment, family history of similar illness in a sibling, and rapid decline in 25OHD levels after treatment was stopped, we suspected monogenic forms of rickets. Genomic DNA was isolated from peripheral blood leukocytes using a QIAamp DNA Blood Mini QIAcube Kit (Qiagen, Hilden, Germany), according to the manufacturer’s instructions. Clinical exome sequencing was performed using the Illumina platform. Libraries were prepared using Twist Human Customized Core Exome Kit (Twist Bioscience, San Francisco, CA, USA). Prepared libraries were sequenced on the Illumina HiSeqX platform (Illumina, San Diego, CA, USA). Up to 75% of the sequenced bases were of Q30 value and libraries were sequenced to a mean depth of >80 to 100X. Sequence alignment and variant calling were performed using the Sentieon software (v201808.01).

A homozygous 10 base pair duplication in exon 1 of the *CYP2R1* gene (NM_024514: c.50_51insTCGGCGGCGC) that results in frameshift and premature truncation of the protein 79 amino acids downstream to codon 18 (p.Leu18ArgfsTer79; ENST00000334636.10) was detected. The variant is not reported in gnomAD (v3.1.1) and 1000genomes databases. The in silico prediction of the variant is damaging by MutationTaster2 (http://www.mutationtaster.org/). The variant was deemed “pathogenic” as per American College of Medical Genetics 2015 criteria (PVS1, PM2, PP1, PP4).

**Figure 3.**
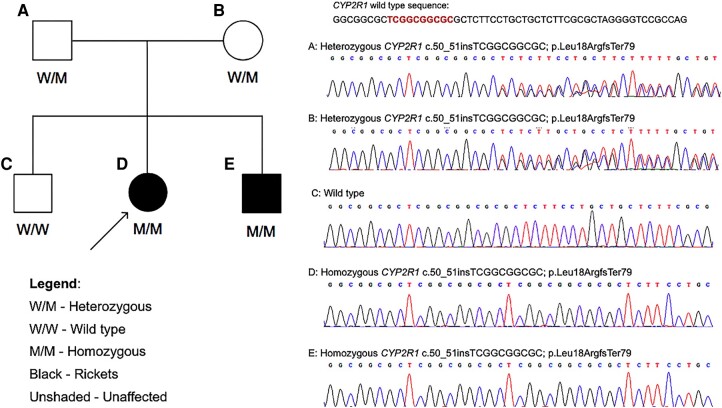
Details of genetic testing of family. The panel on the left shows the pedigree chart of the family. The panel on the right shows the chromatograms from Sanger sequencing of the proband and index relatives for the variant.

The mutation was confirmed in the proband, and all index relatives were evaluated for the presence of the variant by Sanger sequencing [ABI 3730 (Applied Biosystems, CA, USA)]. The variant was present in a homozygous state in the proband and the affected sibling and in the heterozygous state in both parents. An unaffected sibling did not carry the variant (Fig. 3). Biochemical parameters of family members are shown in Table 1.

## Treatment

After confirmation of diagnosis, the proband and her younger brother were given calcium 500 mg/day (approximately 50 mg/kg/day) and cholecalciferol 2000 IU/day. The dose was titrated to 4000 IU/day after 6 months based on biochemical parameters.

## Outcome and Follow-up

They were monitored for clinical, radiological, and biochemical parameters. The serum ALP, 25OHD, and iPTH were maintained in a normal range with this dose. Urinary calcium excretion was monitored to avoid iatrogenic hypercalciuria. There was good radiographic healing on treatment.

## Discussion

We report a novel mutation in *CYP2R1* leading to vitamin-D dependent rickets type 1b (VDDR1b) (OMIM # 600081). To the best of our knowledge, this is the first report of this rare condition from Asia. The patients responded well to treatment with supraphysiological doses of vitamin D.

The condition was first postulated in 1994 by Casella et al in 2 siblings of Nigerian descent who presented with severe rickets despite normal vitamin D intake (8). They had low 25OHD levels with normal 1,25(OH)_2_D and required supraphysiologic doses of vitamin D for healing of rickets. One of these siblings was evaluated by Cheng et al and was found to have a homozygous *CYP2R1* c.296T > C; p.L99P mutation (2). They demonstrated that the L99P mutation resulted in a complete loss of 25-hydroxylase activity in vitro. Levine et al reported the same variant in 2 additional Nigerian families, demonstrating that the variant was common in Nigeria (3).

Tacher et al sequenced the *CYP2R1* gene in 27 children with sporadic rickets and 12 subjects from families in which more than 1 member had rickets (5). No mutations were detected in the 27 children with sporadic rickets, but missense mutations in *CYP2R1* were detected in affected members in 2 of 12 families. They identified the previously known c.296T > C (L99P) mutation and a novel c.726A > C; p.K242N mutation in this cohort and demonstrated by in vitro studies that the variants caused a marked reduction of 25-hydroxylase activity. Heterozygous subjects were less affected than homozygous subjects, and oral administration of vitamin D led to significantly lower increases in serum 25OHD in heterozygous than in control subjects, whereas homozygous subjects showed negligible increases. This was the first demonstration that *CYP2R1* has a semidominant inheritance pattern, with both homozygotes and heterozygotes being affected, with varying severity.

Molin et al reported the condition in 2 families; 1 a nonconsanguineous French family and the other a consanguineous couple of Moroccan descent (6). Analysis of *CYP2R1* identified a novel c.124_138delinsCGG (p.Gly42_Leu46delinsArg) and previously known L99P mutation in homozygous state in both probands of family 1 and 2, respectively. In vitro studies demonstrated a complete loss of function of *CYP2R1* with both variants. Interestingly, adult subjects in the second family were able to sustain near-normal serum levels of calcium, PTH, and ALP despite homozygosity for the nonfunctional p.Leu99Pro allele. This is similar to the natural history of many patients with VDDR2A who are able to maintain normal mineral metabolism in adolescence and adulthood with modest oral calcium supplements, probably due to induction of mechanisms for vitamin D–independent calcium absorption from the intestine, as age advances (9). Alternatively, other CYP enzymes that possess 25-hydroxylase activity may assume greater importance with maturation (6).

Our report increases the number of reported mutations in *CYP2R1*. It also expands its presence to a new geographical area as all previous reports were from either Africa or Europe. In view of the high frequency of consanguinity in some communities and the widespread practice of endogamous mariages in India, it is feasible that the prevalence of autosomal recessive disorders such as VDDR1b is high (10). VDDR1b is likely to be underdiagnosed as the clinical and biochemical features are often indistinguishable from nutritional rickets, except for a requirement of higher doses of vitamin D for successful treatment and a tendency to relapse quickly once treatment is stopped. Also, nutritional rickets may affect multiple members of a family due to common dietary and social factors. Hence, there is a high threshold for seeking a genetic etiology even when there is a positive family history. Furthermore, the condition seems to remit to an extent in adulthood.

Treatment options for VDDR1b include treatment with pharmacological doses of ergocalciferol or cholecalciferol, calcifediol (25OH vitamin D3), or calcitriol. Calcifediol represents the most physiologic option, bypassing the defect in 25 hydroxylation and avoiding toxicity as conversion from 25OHD to 1,25(OH)_2_D will remain regulated. Efficacy of this approach has previously been demonstrated by Molin et al (6). However, availability and cost preclude the universal adoption of this option. Treatment with calcitriol may be hazardous as patients with VDDR1b generally have normal or high levels of 1,25(OH)_2_D and this may lead to hypercalciuria and nephrocalcinosis. We used the first approach and found a good response to treatment targeting normal serum calcium, 25OHD, ALP, and iPTH levels.

## Learning Points

- VDDR1b is a rare, inherited form of rickets that is probably underdiagnosed.
- It exhibits a semidominant inheritance with both homozygotes and heterozygotes being affected, with varying degrees of severity.
- The clinical manifestations generally improve in adulthood, and adults with the condition often do not require treatment.
- Treatment of affected children with supraphysiologic doses of vitamin D targeting normalization of serum 25OHD, ALP, and iPTH is associated with good response.
- Genetic testing of patients with suspected nutritional rickets who have a family history of severe illness requiring higher than usual doses of vitamin D for healing or relapse on stopping treatment may be a good case-finding strategy.

## Data Availability

Original data generated and analyzed during this study are included in this published article.
